# A novel antisense long noncoding RNA regulates the expression of MDC1 in bladder cancer

**DOI:** 10.18632/oncotarget.2861

**Published:** 2014-11-06

**Authors:** Yao Xue, Gaoxiang Ma, Zhensheng Zhang, Qiuhan Hua, Haiyan Chu, Na Tong, Lin Yuan, Chao Qin, Changjun Yin, Zhengdong Zhang, Meilin Wang

**Affiliations:** ^1^ Department of Environmental Genomics, Jiangsu Key Laboratory of Cancer Biomarkers, Prevention and Treatment, Cancer Center, Nanjing Medical University, Nanjing, China; ^2^ Department of Genetic Toxicology, The Key Laboratory of Modern Toxicology, Ministry of Education, School of Public Health, Nanjing Medical University, Nanjing, China; ^3^ Department of Urology, Changhai Hospital, The Second Military Medical University, Shanghai, China; ^4^ Department of Urology, Jiangsu Province Hospital of TCM, Nanjing, China; ^5^ Department of Urology, The First Affiliated Hospital of Nanjing Medical University, Nanjing, China

**Keywords:** antisense lncRNAs, MDC1-AS, expression regulation, bladder cancer

## Abstract

Antisense long noncoding RNAs (lncRNAs) play important roles in regulating the expression of coding genes in post-transcriptional level. However, detailed expression profile of lncRNAs and functions of antisense lncRNAs in bladder cancer remains unclear. To investigate regulation of lncRNAs in bladder cancer and demonstrate their functions, we performed lncRNAs microarray analysis in 3 paired bladder cancer tissues. Further molecular assays were conducted to determine the potential role of identified antisense lncRNA *MDC1-AS*. As a result, a series of lncRNAs were differentially expressed in bladder cancer tissues in microarray screen. In a larger size of samples validation, we found that the expression levels of *MDC1-AS* and *MDC1* was down-regulated in bladder cancer. After over-expression of *MDC1-AS*, increased levels of *MDC1* were observed in bladder cancer cells. We also found a remarkably inhibitory role of antisense lncRNA *MDC1-AS* on malignant cell behaviors in bladder cancer cells EJ and T24. Subsequently, knockdown of *MDC1* revealed that suppressing role of *MDC1-AS* was attributed to up-regulation of *MDC1*. In summary, we have identified a novel antisense lncRNA MDC1-AS, which may participate in bladder cancer through up-regulation of its antisense tumor-suppressing gene *MDC1*. Further studies should be conducted to demonstrate detailed mechanism of our findings.

## INTRODUCTION

Bladder cancer is a common malignancy in urinary system. According to data of Global Cancer Statistics, about 440,000 new cases of bladder cancer are diagnosed while 130,000 patients died from it around the world every year. In China, bladder cancer has the highest incidence in all urinary system tumors and the mortality increased significantly during the past decades [1]. The high recurrence rate of non-invasive bladder cancer [2] and the evident aggressive performance of invasive bladder cancer are the main threaten to patient life. However, the effective therapy for bladder cancer is still deficient because the detailed mechanism underlying bladder cancer is unclear. Therefore, intensive study into the molecular mechanism will promote clinical treatment of bladder cancer.

With the advance of high-throughput transcriptome analyses, most of the human genome has been found to be transcribed into noncoding RNAs. Different from the conventional coding genes, which exert their biological functions by translated into protein molecules, noncoding RNAs play important roles in regulation of various biological process in form of RNA, and do not have the capability to coding proteins [3]. Among the noncoding RNAs, a group of long noncoding RNAs (lncRNAs) with a length of >200 nucleotides have been highlighted in virtue of their large amount and the prominent part they played in intracellular regulation network [4-6]. Recent studies have demonstrated that lncRNAs were closely involved in carcinogenesis and can be used as the potential biomarkers of cancer [7-10]. Besides, several lncRNAs were identified to influence occurrence or progress of bladder cancer, e.g. *ncRNA* [11], *UBC1* [12], *GAS5* [13], etc.. However, most of the role of lncRNAs in bladder cancer remains unknown yet.

According to their relative location to adjacent coding genes, lncRNAs can be roughly divided into 5 categories, i.e. antisense lncRNAs, sense lncRNAs, intronic lncRNAs, bidirectional lncRNAs and intergenic lncRNAs [14]. Among them, antisense lncRNAs have been recognized to regulate expression of corresponding coding genes in post-transcriptional level [15] and therefore participate in carcinogenesis by regulation of oncogenes as well as anti-oncogenes. For instance, antisense transcript of coding gene *PCNA*, *PCNA-AS1*, was recently shown to increase *PCNA* mRNA stability and promote hepatocellular tumor growth [16].

Mediator of DNA damage checkpoint protein 1 (MDC1) was an important mediator of the repair of double-strand breaks (DSB). It acts as a strong tumor suppressor through its DNA damage repair function and may be involved in carcinogenesis of bladder cancer. However, antisense transcript of *MDC1*, named *MDC1-AS* in the present study, was a novel unknown lncRNA. Studies on the biological function of *MDC1-AS* and its role in carcinogenesis have not been reported yet.

To investigate the dysregulated lncRNAs in bladder cancer and demonstrate their biological roles, we conducted lncRNA microarray assay using 3 paired bladder cancer tissues. For the identified dysregulated lncRNAs, we performed preliminary screening and selected *MDC1-AS* as the main subject of the present study. The following molecular assays demonstrated a tumor-suppressor role of this novel antisense lncRNA.

## RESULTS

### Difference of lncRNA expression profile between bladder cancer tissues and non-cancer tissues

Fold change greater than 2 and *P* value less than 0.05 between cancer tissues and non-cancer tissues were set as the criteria in filtering differently expressed lncRNAs. In summary, there were 562 up-regulated and 672 down-regulated lncRNAs in bladder cancer tissues compared with adjacent normal tissues, including 152 antisense lncRNAs, 89 sense lncRNAs, 158 intronic lncRNAs, 58 bidirectional lncRNAs and 771 intergenic lncRNAs.

**Figure 1:**
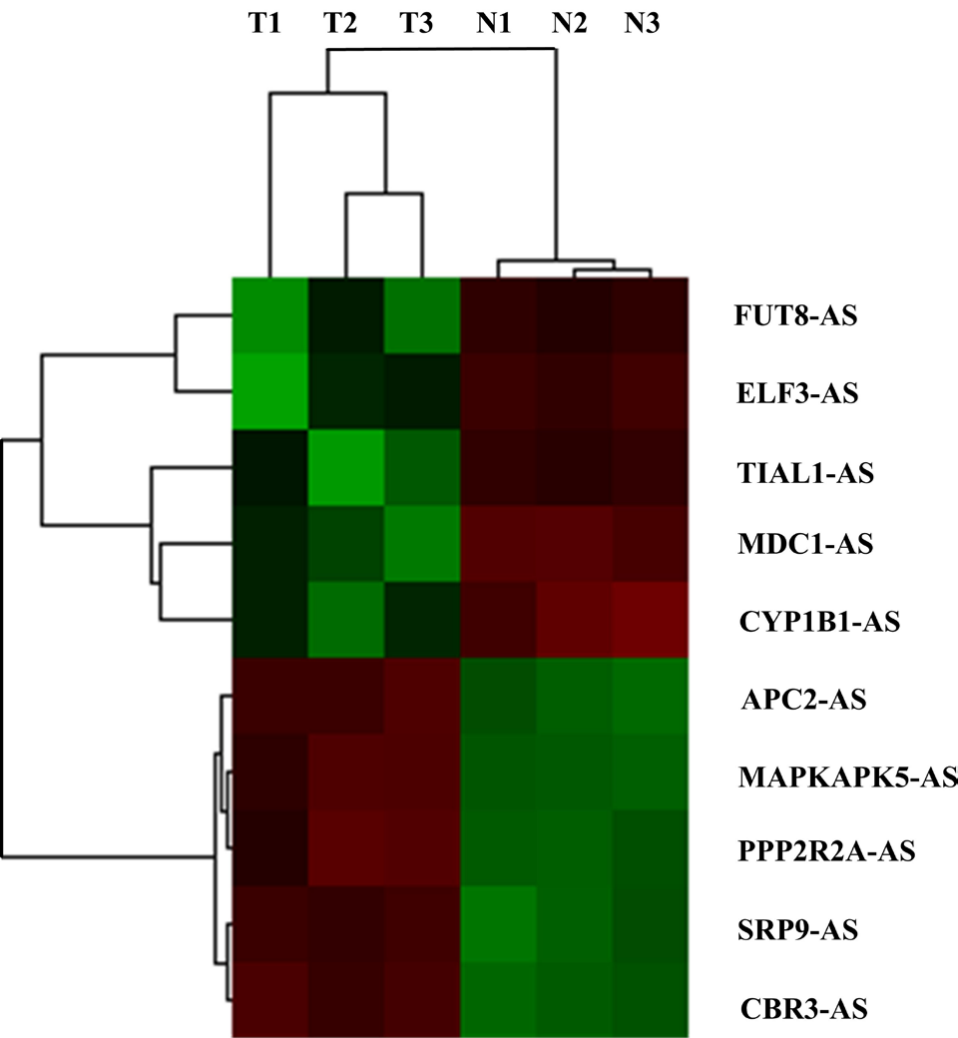
Hierarchical clustering analysis on the most 10 significantly dysregulated cancer-related antisense lncRNAs resulted from microarray assay All the antisense lncRNAs were named according to their adjacent cancer-related coding genes. T1, T2, T3 and N1, N2, N3 represented for cancerous and paired non-cancerous tissues of samples BC1, BC2 and BC3, respectively.

Results of hierarchical clustering analysis on the most significantly dysregulated cancer-related antisense lncRNAs are shown in Figure 1. Through further literature search on the adjacent coding genes, we found that coding gene *MDC1* was more deeply involved in carcinogenesis than the others. It was an important participant in DNA damage repair process with established tumor-suppress function. Therefore, in the following study, we focused on antisense lncRNA of *MDC1, MDC1-AS* (transcript number ENST00000442150), and performed a series of molecular biological assays to deeply investigate role of *MDC1-A*S in bladder cancer.

### Dysregulation of *MDC1-AS* and *MDC1* expression level

**Figure 2:**
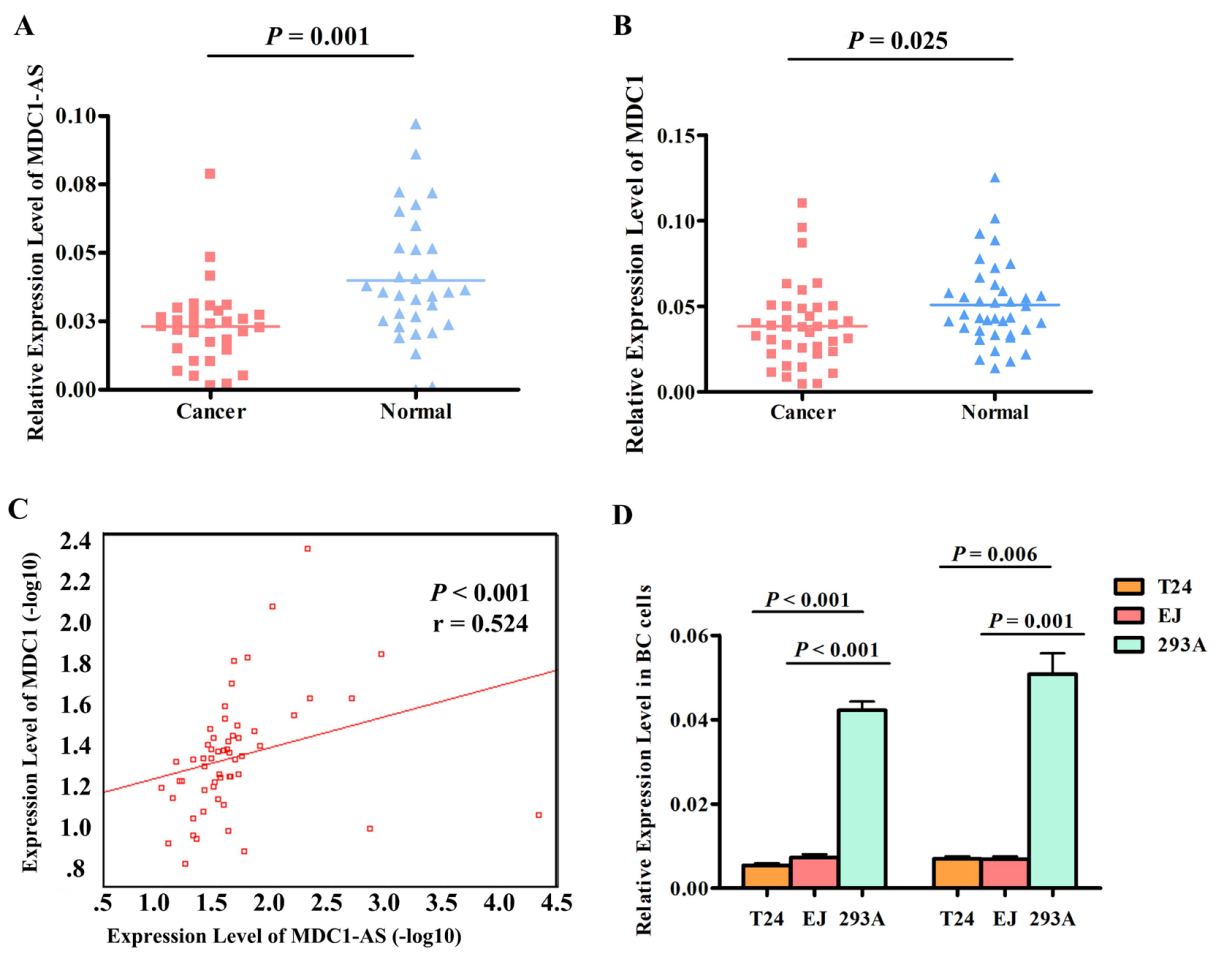
Dysregulation of *MDC1-AS* and *MDC1* expression level (A) *MDC1-AS* levels in cancerous tissues were significantly lower than those in the noncancerous tissues. (B) *MDC1* was down-regulated in bladder cancerous tissues, compared with that in adjacent normal tissues. (C) Expression levels of *MDC1-AS* and *MDC1* in tissues were significantly correlated in a positive direction. (D) Both *MDC1-AS* and *MDC1* were decreased in bladder cancer cells T24 and EJ, compared with those in normal cell line 293A.

Thirty-eight pairs of bladder cancer tissues and adjacent noncancerous tissues were used to confirm the aberrant expression level of *MDC1-AS* obtained from microarray assay by RT-PCR. It should be stated that *MDC1-AS* RT-PCR data were only available in 32 pairs of tissues because the absolute quantity of *MDC1-AS* is very few and the transcripts were not stable. Consistently, *MDC1-AS* levels in cancerous tissues were evidently lower than those in the noncancerous tissues (*P* = 0.001, as shown in Figure 2A).

In addition to *MDC1-AS*, levels of coding gene *MDC1* were also detected in the 38 pairs of tissues. AS shown in Figure 2B, *MDC1* was also down-regulated in bladder cancer tissues (*P* = 0.025), which was concordant with *MDC1-AS*.

Spearman's correlation analysis was performed to determine the correlation between *MDC1-AS* and *MDC1* in statistical level. As a result, expression levels of *MDC1-AS* and *MDC1* in tissues were significantly correlated in a positive direction (*P* < 0.001, Figure 2C), indicating a potential regulating role of *MDC1-AS* on the coding gene *MDC1*.

Furthermore, expression levels of *MDC1-AS* and *MDC1* were also detected in two bladder cancer cell lines T24 and EJ. Consistent with the dysregulated tendency in tissues, both *MDC1-AS* and *MDC1* were decreased in bladder cancer cells, compared with those in normal cell line 293A (Figure 2D).

Down-regulation of coding gene *MDC1* was further confirmed in protein level. As a result, bladder cancer tissues showed observably reduced *MDC1* protein levels than noncancerous tissues.

In addition, we analyzed the association between *MDC1-AS* and *MDC1* expression levels and clinical factors of bladder cancer patients. However, no significant association was found in this analysis (data not shown).

### Coding capability prediction and subcellular location of *MDC1-AS*

Results of coding capability prediction using *MDC1-AS* sequence suggested that *MDC1-AS* did not possess the ability to code any protein (Supplementary Figure 1, coding probability of *MDC1-AS* was 0.138).

EJ cells were separated into nuclear and cytoplasmic fractions to determine the cellular location of both *MDC1-AS* and *MDC1*. As presented in Supplementary Figure 2, we found that both *MDC1-AS* and *MDC1* were exist primarily in the nuclear fraction. We attribute this phenomenon to the DNA repair function of *MDC1*. Besides, the same location of *MDC1-AS* and *MDC1* indicated potential interrelations between them.

### Regulating role of *MDC1-AS* on *MDC1* expression level

**Figure 3:**
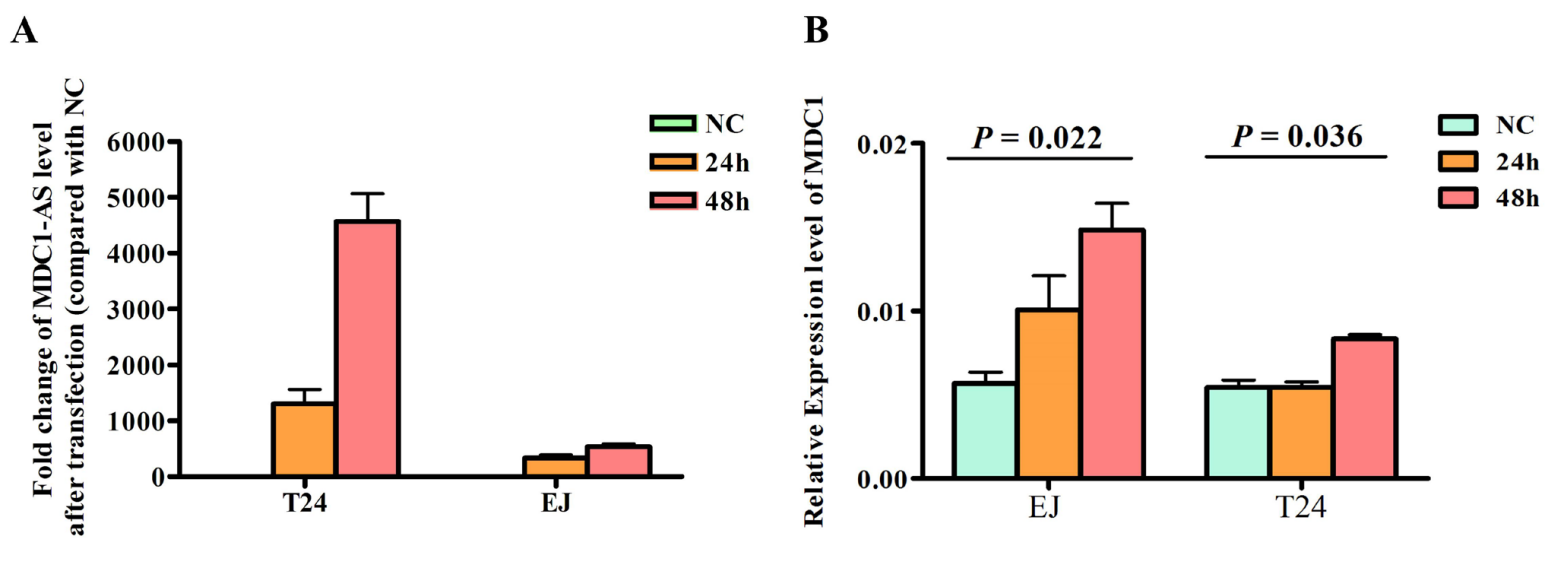
Regulating role of *MDC1-AS* on *MDC1* expression level (A) Over-expression of MDC1-AS in bladder cancer cells T24 and EJ with transfection of vectors, value of NC group was set as 1 and not presented in this figure. (B) Levels of MDC1 mRNA in cells were significantly increased after transfected with MDC1-AS over-expressed vector for 48h.

To investigate whether *MDC1-AS* regulate expression level of protein coding gene *MDC1*, we enhanced *MDC1-AS* expression by transfecting an expression vector containing *MDC1-AS* into T24 and EJ cells, using pcDNA3.1 empty vector as negative control. Twenty-four hours and 48 h after transfection, levels of *MDC1-AS* and *MDC1* in these two cell lines were detected. Results revealed that *MDC1-AS* was significantly over-expressed (Figure 3A), indicating a success in our transfection and over-expression of *MDC1-AS* in bladder cancer cells.

With the over-expression of *MDC1-AS*, up-regulation of coding gene *MDC1* was also observed in both RNA (Figure 3B) and protein levels. Besides, this up-regulation was repeatedly confirmed in two cell lines, suggesting that *MDC1-AS* may exert a positive effect on expression of *MDC1*.

### Inhibitory role of *MDC1-AS* on cell proliferation and colony formation

**Figure 4:**
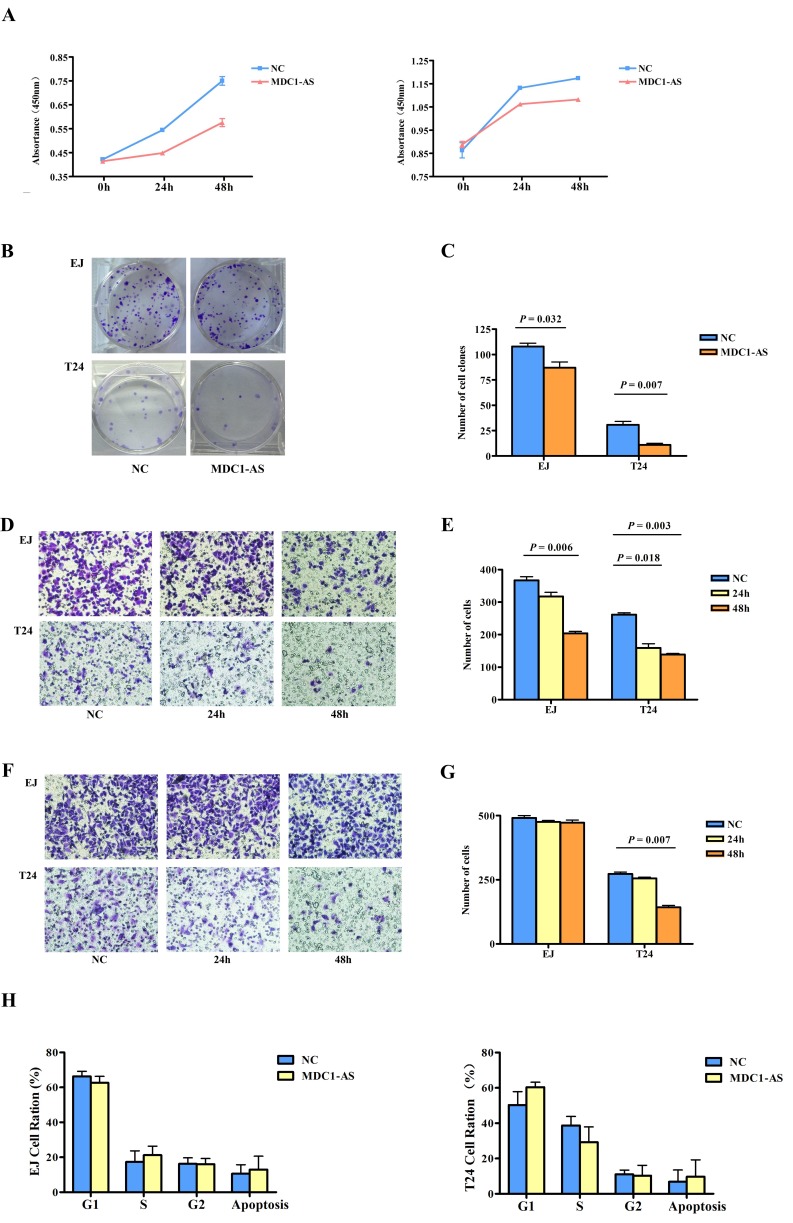
Inhibitory role of *MDC1-AS* on malignant cell behavior (A) Proliferation of bladder cancer cells EJ (left) and T24 (right) were significantly decreased after *MDC1-AS* expression was enhanced for 24h and 48h. (B) Colony formation of EJ and T24 cells was evidently suppressed following *MDC1-AS* over-expression and (C) Statistical chart of clone number. (D) Migration of EJ and T24 cells were decreased after over-expressing of MDC1-AS and (E) Number of migrated cells. (F) Significant impairment of T24 cell invasion after transfected with *MDC1-AS* for 48h and (G) Number of invaded cell. (H) Distribution of cells in each cell cycle phase was not changed after MDC1-AS over-expressing.

The significant down-regulation of *MDC1-AS* in bladder cancer tissues and cells prompted us to explore the potential biological functions of *MDC1-AS* in carcinogenesis. Firstly, CCK-8 assay showed that proliferation of bladder cancer cells EJ and T24 were remarkably inhibited after *MDC1-AS* expression was enhanced for 24h and 48h, compared with those transfected with NC vectors (Figure 4A and Supplementary Table 2, *P* < 0.05 in all the experimental groups).

As colony formation was another important indicator for the viability of cells, we further detect effect of *MDC1-AS* on cells colony formation. In accordance with t he proliferation assay, observations in colony formation assay also indicated that EJ and T24 cells clonogenic survival was evidently suppressed following over-expression of *MDC1-AS* (Figure 4B and 4C, *P* = 0.032 for EJ cells and *P* = 0.007 for T24 cells).

### Inhibitory role of *MDC1-AS* on cell migration and invasion

Following the verification of inhibitory role of *MDC1-AS* on bladder cancer cells viability, we further investigate whether this lncRNA participant in cells migration and invasion. Compared with EJ and T24 cells transfected with NC vector, cells over-expressing *MDC1-AS* for 48h showed significantly decreased ability in migration (Figure 4D and 4E, *P* = 0.006 for EJ cells and *P* = 0.003 for T24 cells). Besides, the suppression effect on migration was also observed in T24 cells after 24h of transfection (*P* = 0.018).

Subsequently, in the evaluation of cells invasion, T24 cells exhibited significant impairment of invasion ability after transfected with *MDC1-AS* for 48h (Figure 4F and 4G, *P* = 0.007). However, no significant difference was observed in EJ cells.

In addition to the malignant behaviors mentioned above, we also analyzed cell cycle progression in cells treated with *MDC1-AS* and NC vectors. However, there were no distinct changes in the distribution of cells in each cell cycle phase, indicating that the inhibitory effect of *MDC1-AS* on cell proliferation was irrelevant to cell cycle arrest (shown in Figure 4H)

### Knockdown of *MDC1* expression by siRNA

In order to determine whether the observed inhibitory effects of *MDC1-AS* was the consequence of its up-regulation of coding gene *MDC1*, knockdown of *MDC1* expression was achieved by siRNA interference. RT-PCR assay of the interfered cells showed that *MDC1* level was markedly decreased, except for si-MDC1-3 (Supplementary Figure 3). In the following study, siMDC1 with the highest inhibition ratio up to 80% (i.e. si-MDC1-1, *P* = 0.025) was selected. Malignant phenotypes were monitored repeatedly in EJ and T24 cells with both *MDC1-AS* over-expressing and *MDC1* knockdown.

### Repeating observation on cell proliferation, colony formation, migration and invasion

**Figure 5:**
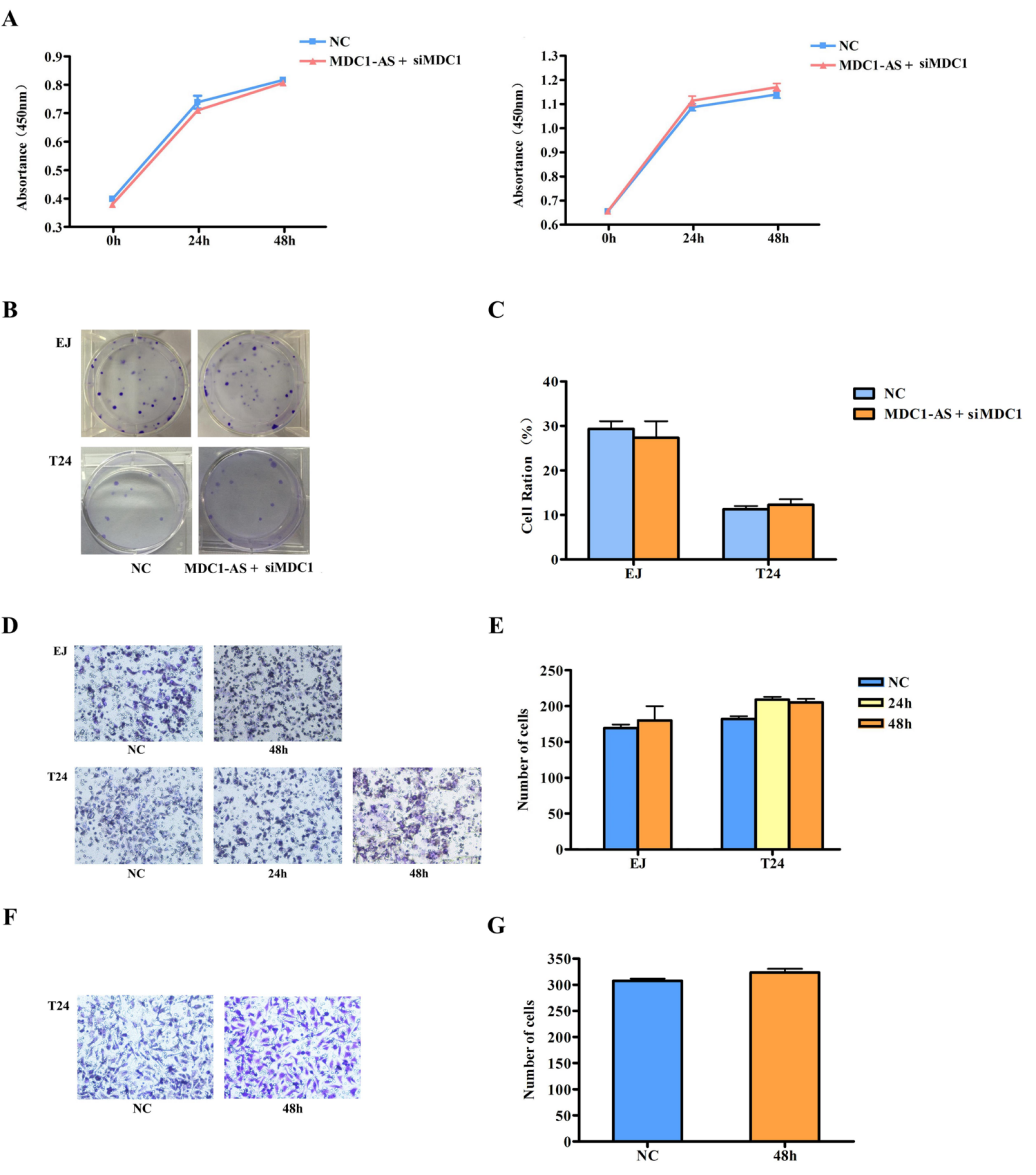
Repeating observation on malignant cell behavior after co-transfected with *MDC1-AS* and siMDC1 Inhibitory role of MDC1-AS on malignant cell phenotype, i.e. (A) proliferation (the left was EJ cell and the right was T24 cell); (B) and (C) colony formation; (D) and (E) migration; (F) and (G) invasion, was diminished after MDC1 was knockdown.

In the repeated CCK-8 assay, we found that inhibitory role of *MDC1-AS* on bladder cancer cells proliferation was evidently weakened with co-transfection of siMDC1. As shown in Figure 5A and Supplementary Table 3, there was no significant change in proliferation ratio of treated EJ and T24 cells, compared with the NC groups.

Similarly, suppression on cell colony formation was also attenuated by siMDC1, that is, *MDC1-AS* did not have the ability to inhibit colony formation of bladder cancer cells any longer after *MDC1* was knockdown (Figure 5B and 5C).

Furthermore, observation on cell migration and invasion were also repeated and time points were selected according to time finding significant differences in the previous study. As a result, no differences were found between co-transfected cells and cells with NC vector (Figure 5D - 5G), indicating that siMDC1 also diminishes suppressing role of *MDC1-AS* on cells migration and invasion.

## DISCUSSION

Human Genome Project has revealed that most of the transcriptional output of the whole genome was lncRNAs, which were far more than the coding transcripts and short noncoding RNAs [3]. The huge quantity also indicated fundamental functions of lncRNAs. Studies have demonstrated that an important category of lncRNAs was transcripts from the opposite strand to a protein coding gene, called antisense lncRNAs [15, 17]. Along with the development of relevant researches, antisense lncRNAs were suggested to function as epigenetic regulators of transcription through various mechanisms [17-18]. They have been identified to hybridize with the coding strand or their antisense transcripts, with the consequence of interfering with transcription or stability of mRNA [15]. Several cancer-related genes, such as p15 [19] p53 [20] and Myc [21] have already been demonstrated to be regulated by their antisense lncRNAs. However, determine the detailed mechanism of the large number of antisense lncRNAs with unknown function was still a huge challenge to our understanding of the regulation on human genome.

In the present study, microarray analysis of lncRNAs expression revealed a set of lncRNAs expressing differently between bladder cancer tissues and adjacent normal tissues, indicating the participation of lncRNAs in carcinogenesis of bladder cancer. This result further promoted us to detect deeply into the detailed functions of the abnormally expressed lncRNAs.

MDC1 is an important mediator of the repair of double-strand breaks (DSB) with an amino-terminal forkhead-associated (FHA) domain and a tandem repeat of breast cancer susceptibility gene-1 carboxy terminus (BRCT) domains [22]. In response of DSB damage, MDC1 was activated by ataxia telangiectasia mutated (ATM) protein kinase and recruit DNA damage repair (DDR) factors to DSB sites [23]. It is well known that unrepaired DSB will result in genome instability and promote apoptosis or oncogenesis [24]. Therefore, involvement of DDR factors, including MDC1, in the mechanism of carcinogenesis has aroused great concern. For instance, MDC1-knockout mice presented susceptibility to cancer [25] and lack of MDC1 expression has been observed in various human cancers [26]. In general, MDC1 acts as a strong tumor suppressor through its DDR function.

Given the vital role of antisense lncRNAs on regulating their neighboring coding genes and the definite tumor-suppressing role of MDC1, we focused on *MDC1-AS* in the following study and hypothesized that this antisense lncRNA may take part in bladder cancer through regulation of *MDC1*.

Firstly, RT-PCR detection in a larger size of tissues verified the significant down-regulation of lncRNA *MDC1-AS* and the reduction of *MDC1-AS* expression was also observed in bladder cancer cells EJ and T24, suggesting that *MDC1-AS* may act as a tumor suppressor in bladder cancer. Besides, expression of coding gene *MDC1* in bladder cancer tissues also showed similar trend with *MDC1-AS*, compared with those in non-tumor tissues. This observation was consistent with previous studies reporting decreased *MDC1* level in cancerous tissues [26-27] and provided further support for tumor suppressing role of *MDC1*. In Spearman's correlation analysis, evident correlation was found between expression levels of *MDC1-AS* and *MDC1*. Therefore, lncRNA *MDC1-AS* was likely to exert positive regulatory effect on its neighboring coding gene *MDC1*. However, we didn't observe significant associations between MDC1-AS and MDC1 expression level and clinical factors of bladder cancer, we speculated that this may be due to relatively small size of our tissue samples. Further bioinformatic prediction revealed that sequence of *MDC1-AS* did not code for protein, this result provided further support for the characteristic of *MDC1-AS* as a long noncoding RNA, in addition to the database information. (http://asia.ensembl.org/Homo_sapiens/Transcript). Subsequently, elevated expression level of *MDC1* was observed in *MDC1-AS* over-expressing cells. These findings suggested that *MDC1-AS* can up-regulate coding gene *MDC1* in both mRNA and protein level, thus verified the assuming role of *MDC1-AS* mentioned above.

After demonstrating regulatory role of *MDC1-AS* on tumor suppressing gene *MDC1*, we detected the influence of it on bladder cancer cells. In a series assays on malignant cell behaviors, cancer cells over-expressing *MDC1-AS* exhibited significant decreases in proliferation, colony formation, migration and invasion. The observed reduction in malignant cell behavior provided evidence for the tumor suppressing role of *MDC1-AS*, which were consistent with our conjecture. Therefore, the significant down-regulation of *MDC1-AS* observed in bladder cancer tissues and cells can be interpreted: cells in cancer tissues showed lower level of *MDC1-AS*, thus their malignant phenotypes were more evident than those in normal tissues.

At this point, our study has found an up-regulating effect of *MDC1-AS* on coding gene *MDC1*, and distinct suppressing roles of this lncRNA on cancer cells. As *MDC1* was an identified tumor-suppressing gene, we wondered whether the inhibitory role of *MDC1-AS* on bladder cancer cells was attribute to up-regulation of *MDC1*. Therefore, we interfered intracellular expression of *MDC1* to investigate on this issue. As a result, cells co-transfected with *MDC1-AS* and siMDC1 presented similar malignant behavior with NC group, suggesting that inhibitory role of *MDC1-AS* was remarkably diminished when *MDC1* was knockdown. Taken together, it stood to reason that *MDC1-AS* exhibit an inhibitory role on bladder cancer cells through its up-regulation of a tumor suppressing gene, *MDC1*.

Recently, more and more studies have investigated into biological effects of genetic variants on lncRNAs. Researchers have found that single nucleotide polymorphisms (SNPs) located in lncRNAs may be associated with cancer risk and modify expression level or biological functions of the lncRNA [28-30]. Therefore, further studies focused on the effects of SNPs in *MDC1-AS* will be needed to clarify the role of genetic variants on lncRNAs in carcinogenesis of bladder cancer.

There was a limitation of our study. Although we demonstrated the regulation role of *MDC1-AS* in *MDC1* expression, the detailed mechanism underlying this regulation was still not known. According to previous study reported by Faghihi *et al*., antisense lncRNA may elevate expression level of coding genes through increasing of mRNA stability [31]. Investigation on this issue will be conducted in further studies.

In conclusion, we have identified *MDC1-AS* as a novel antisense lncRNA through lncRNA microarray approach. This lncRNA was down-regulated in bladder cancer tissues, and exerted a positive effect on expression of coding gene *MDC1*. In addition, *MDC1-AS* significantly inhibited malignant phenotype of bladder cancer cells, through up-regulation of *MDC1*. Further studies should be conducted to investigate the detailed mechanism of our findings.

## MATERIAL AND METHODS

### Patient specimens and clinical assessments

In general, 41 pairs of bladder cancer tissues and adjacent normal bladder tissues were collected in the present study. All of the samples were obtained from the First Affiliated Hospital of Nanjing Medical University and Jiangsu Province Hospital of TCM in Nanjing, China. All samples were frozen in liquid nitrogen immediately after surgical resections. The hematoxylin and eosin (H&E) stained sections prepared using the cancerous and normal tissues were histologically confirmed by the pathologists. Detailed information of the 3 cases randomly selected to be analyzed in microarray platform is shown in Supplementary Table 1. The informed consent was obtained from all the participants and procedures used in this study were approved by the institutional review boards of Nanjing Medical University.

### RNA extraction

Total RNA was extracted from all the tissues samples as well as cancer cells using Trizol reagent (Invitrogen, CA, USA) according to the manufacturer's instruction. NanoDrop ND-1000 spectrophotometer (OD 260 nm, NanoDrop, Wilmington, DE, USA) was used to assess the quantity of RNA, and standard denaturing agarose gel electrophoresis was used to assess RNA integrity.

### LncRNA microarray and data analysis

Genome-wide lncRNA expression assay was performed using Human LncRNA Microarray V2.0, which was manufactured by Arraystar Inc (MD, USA). This microarray covered more than thirty-three thousand lncRNAs identified in human genome. Raw signal intensities of the microarray were normalized by GeneSpring GX v12.0, and low intensity LncRNAs were filtered. Passing Volcano Plot filtering was used to identify differentially expressed LncRNAs with statistical significance between the two groups. The microarray hybridization and collection of expression data were performed by KangChen Bio-tech, Shanghai, China.

### Hierarchical clustering analysis

For the important regulating role of antisense lncRNAs on their adjacent coding genes, biological functions and their role in carcinogenesis of the adjacent coding genes were retrieved in literatures and we assumed that those lncRNAs with cancer-related adjacent coding genes were most likely to be involved in the occurrence of malignancies. Unsupervised hierarchical clustering analysis of the cancer-related antisense lncRNAs was described in *Supplementary Methods*.

### Quantitative real-time PCR (qRT-PCR)

cDNA was synthesized from total RNA using M-MLV reverse transcriptase (Invitrogen) according to the manufacturer's protocol. qRT-PCR with SYBR Green assays (TaKaRa Biotechnology, Dalian, China) was used to determine expression level of selected lncRNA *MDC1-AS* and its adjacent coding gene *MDC1*. A detailed description of qRT-PCR was provided in *Supplementary Methods*.

### Transfection of *MDC1-AS* overexpression vector

The vectors expressing *MDC1-AS* were prepared by amplifying full length of complementary DNA encoding *MDC1-AS* and the amplified fragments were then cloned into pcDNA 3.1 vector (Invitrogen, Carlsbad, CA, USA). The amplified fragments were then sequenced to confirm that there were no errors in nucleotides. Bladder cells EJ and T24 were transiently transfected with the *MDC1-AS* overexpression plasmid using Lipofectamine 2000 (Invitrogen, Carlsbad, CA, USA) transfection reagent according to the protocols. The pcDNA3.1 empty vector was used as negative control (NC).

### Observation about malignant behaviors of cancer cells

We conducted a series of assays to determine the effects of lncRNA MDC1-AS on the malignant behaviors of bladder cancer cells, e.g. proliferation, colony formation and migration. Detailed assay conditions were described in *Supplementary Methods*.

### RNA interference

To further detect whether the inhibitory effect of *MDC1-AS* on cancer cells was attribute to the upregulation of coding gene *MDC1*, artificial downregulation of *MDC1* was achieved by small interfering RNA (siRNA). All the 3 siRNAs were targeting nonoverlapping regions of *MDC1* cDNA and therefore only knockdown the targeted *MDC1* transcript, not *MDC1-AS*. After measurement of the interference efficiency, si-MDC1-1 (named siMDC1 in the present study) with the highest efficiency was selected for the following study. Sequence of siMDC1 was provided in *Supplementary Methods*.

### Statistical analysis

Independent t-test was used to compare expression level of lncRNAs obtained by microarray as well as continuous data generated in the following functional studies between two groups. Spearman's correlation analysis was used to detect the correlationship between level of *MDC1-AS* and *MDC1* in biological tissues. All statistical analyses were performed by SPSS for v.13.0 (SPSS, Chicago, IL). P-values of less than 0.05 were considered significant.

There were some other methods and detailed informations listed in Supplementary Methods.

### Funding

This work was partly supported by National Natural Science Foundation of China (81473050, 81230068, 81373091, 81202268 and 81102089), Natural Science Foundation of Jiangsu Province (BK2011773, and BK2011775), the Key Program for Basic Research of Jiangsu Provincial Department of Education (12KJA330002 and 11KJB330002), Jiangsu Provincial Six Talent Peaks Project (2012-SWYY-028, and 2012-WSN-30), The research project of Jiangsu Province of TCM (y13031), Specialized Research Fund for the Doctoral Program of Higher Education (20123234110001), Jiangsu Provincial Science and Technology Innovation Team, and the Project Funded by the Priority Academic Program Development of Jiangsu Higher Education Institutions (Public Health and Preventive Medicine).

## SUPPLEMENTARY FIGURES AND TABLE


