# Supplementary material for: A novel antisense long noncoding RNA regulates the expression of MDC1 in bladder cancer
Source: Oncotarget. 2014 Nov 6;6(1):484–93. doi: 10.18632/oncotarget.2861 (PMC4381609; doi:10.18632/oncotarget.2861)
Supplement: Supplementary file 1 [file oncotarget-06-484-s001.pdf]

A novel antisense long noncoding RNA regulates the expression of MDC1 in bladder cancer

Supplementary Material

Hierarchical clustering analysis

Antisense lncRNAs predicted to participate in carcinogenesis were ranked by their fold change of expression between cancer tissues and normal tissues. Subsequently, unsupervised hierarchical clustering analysis was performed to obtain a general overview of expression profile on the most significantly dysregulated cancer-related antisense lncRNAs using Cluster/TreeView program.

Quantitative real-time PCR (qRT‑*PCR)*

Glyceraldehyde 3-phosphate dehydrogenase (*GAPDH*) was used as an internal control. All the assays were performed using ABI 7300 system (Applied Biosystems, CA, USA). Primers for qRT-PCR were shown in Supplementary Table 4. For quantitative results, the expression of each lncRNA was calculated according to fold change using 2-ΔΔCt methods.

Prediction of lncRNA coding capability

An online method Coding Potential Assessment Tool (CPAT) was used to identify coding capability of the transcript sequence of *MDC1-AS*. CPAT was a novel alignment-free method, which can built a logistic regression model by four characteristics of a given sequence: open reading frame coverage, open reading frame size, hexamer usage bias and Fickett TESTCODE statistic. FASTA-formatted data of

MDC1-AS sequences was inputted into the website and CPAT generated coding probability, the cutoff of which was 0.364 (coding probability > 0.364 indicates coding sequence).

Subcellular fractionation

To further detect the cellular location of lncRNA *MDC1-AS* and its neighboring coding gene *MDC1*, cytosolic and nuclear fractions of bladder cancer cell lines EJ and T24 were isolated and collected separately by PARIS kit AM1921 (Ambion, TX, USA) as the manufacturer’s instructions. After that, total RNA was extracted from the collections of both cytoplasm and nucleus and cDNA was synthesized for the evaluation of *MDC1-AS* and *MDC1* expression.

Western blot analysis

After transfected with *MDC1-AS* or NC vectors, total cells were lysed in RIPA buffer mixed with 0.5% PMSF. They were then incubated on ice and blended completely for 30 min. After centrifugation at 12,000 g for 15 min, the supernatants were collected as protein samples. The protein samples were separated in 10 % SDS–PAGE gels, transferred onto PVDF membranes (Millipore, Billerica, MA, USA), and then blocked with 5 % non-fat milk for 2 h. Then the PVDF membranes were incubated with mouse monoclonal antibody to MDC1 (Abcam, Hongkong) at 4 °C overnight, and with anti-mouse secondary antibody at room temperature for 2 h. After washed with TBST for 2 h, the membranes were visualized with enhanced chemiluminescence.

β-actin was used as an endogenous protein for normalization.

Cell proliferation assay

Approximately 5.0 × 10^3 EJ or T24 cells were plated in 96-well plates after transfected with *MDC1-AS* overexpression or NC vectors. Twenty-four hours and 48 h later, cell proliferation was measured using the Cell Counting Kit-8 (Beyotime, China) according to the manufacturer's protocol. The absorbance was assessed at 450 nm using the Infinite M200 spectrophotometer (Tecan, Switzerland). All experiments were performed in triplicate. The curves of cell proliferation were plotted using the absorbance at each time point.

Cell colony formation assay

After transfection, about 50 cells were plated in the culture dish with a diameter of 10cm. Then they were cultured in RPMI 1640 with 10% fetal calf serum for 14 days. Cell clones were the counted using ImageJ software v2.1.4 (National Institutes of Health, Bethesda, USA). Three independent experiments were performed in parallel.

Cell migration assay

To detect the effect of *MDC1-AS* on EJ and T24 cell migration, we performed transwell assay. Twenty-four hours and 48 h after transfection, cells were harvested and 1 × 10^5 cells were seeded into the upper chamber of the trans-well (pore size, 8 μm; Millpore) with 100 μl of serum-free medium. Medium containing 10% FBS were

added to the lower chamber. After 12 h of incubation at 37˚C, cells remaining on the upper membrane were removed by wiping with a cotton swab. Cells that migrated through the membrane were fixed in 95% methanol for 20 min, stained in 0.1% crystal violet for 15 min and then the membrane was subjected to microscopic inspection (original magnification, × 200). Cell migration was measured by counting cells in five fields per membrane and experiments were independently repeated three times.

Cell invasion assay

Similar to migration assay, invasion assay was also done in transwell chamber. One day before cells seeded on the upper chamber, all the trans-wells were spread by Matrigel (Becton Dickinson Labware, Bedford, MA). The following process of cells seeding was the same as that in migration assay. After incubation, cells remained as well as the Matrigel coating on the upper surface of the filter was wiped off. Finally, invaded cells were observed and counted following the steps described in migration assay.

Cell cycle analysis

Forty-eight hours after transfection, cells were harvested and fixed in 70% pre-cooled ethanol. Then they were stained with 50 μg/ml of propidium iodide (PI) and their DNA contents were analyzed by flow cytometry on a FACS Calibur system (Beckman Coulter, San Jose, CA). The percentage of cells in each cell cycle phase was used as indications of cell cycle progression.

RNA interference

Sequence of siMDC1 was GCGGCUACAUAUCUUUAGUTT (sense) and ACUAAAGAUAUGUAGCCGCTT (antisense). Subsequently, observation on malignant phenotypes of cells co-transfected with *MDC1-AS* and *siMDC1* was performed.

Supplementary Table 1: Clinical characteristics of 3 bladder cancer cases selected in microarray analysis

ID	Age	Gender	Grade	Stage
BC1	84	Female	3	Ⅲ
BC2	76	Male	1	Ⅰ
BC3	58	Male	3	Ⅱ

Supplementary Table 2: Proliferation of EJ and T24 cells over-expressing MDC1-AS detected by CCK-8 assay

Groups	0 h		24 h		48 h	
	OD (mean ± SD)	*P*	OD (mean ± SD)	*P*	OD (mean ± SD)	*P*
EJ-NC	0.42 ± 0.01	0.270	0.54 ± 0.01	< **0.001**	0.75 ± 0.03	**0.002**
EJ-MDC1-AS	0.41 ± 0.01		0.45 ± 0.01		0.58 ± 0.03	
T24-NC	0.86 ± 0.06	0.519	1.13 ± 0.01	**0.001**	1.17 ± 0.01	**0.002**
T24-MDC1-AS	0.89 ± 0.02		1.06 ± 0.01		1.08 ± 0.01	

Supplementary Table 3: Repeating CCK-8 assay on proliferation of EJ and T24 cells co-transfected with MDC1-AS and siMDC1

Groups	0 h		24 h		48 h	
	OD (mean ± SD)	*P*	OD (mean ± SD)	*P*	OD (mean ± SD)	*P*
EJ-NC	0.40 ± 0.01	0.071	0.74 ± 0.04	0.304	0.82 ± 0.02	0.391
EJ-MDC1-AS-siMDC1	0.38 ± 0.01		0.71 ± 0.01		0.81 ± 0.01	
T24-NC	0.65 ± 0.02	0.904	1.09 ± 0.03	0.345	1.14 ± 0.03	0.237
T24-MDC1-AS-siMDC1	0.66 ± 0.01		1.11 ± 0.04		1.17 ± 0.03	

Supplementary Table 4: Sequences of primers used in RT-PCR assay

	Sense (5'-3')	Antisense (5'-3')
MDC1-AS	TCCCAGATGTGCCAAAGTCAG	AGCAACCCCAGTTGTCATTC
MDC1	GCAGCTTCCAGACAACAG	TACCCATGACTTTATCCACA
GAPDH	AAGGTGAAGGTCGGAGTCAAC	GGGGTCATTGATGGCAACAATA

CPAT
Coding Potential Assessment Tool

Calculator User Guide Feedback Source Code

Result for species name : hg19 with job ID :1383641272							
Data ID	Sequence Name	RNA Size	ORF Size	Ficket Score	Hexamer Score	Coding Probability	Coding Label
0	HG19_CT_USERTRACK_3545_	738	315	0.8856	0.0187309964456	0.13825719994722	no

Supplementary Figure 1: Coding capability prediction suggested that *MDC1-AS* did not possess the ability to code any protein.

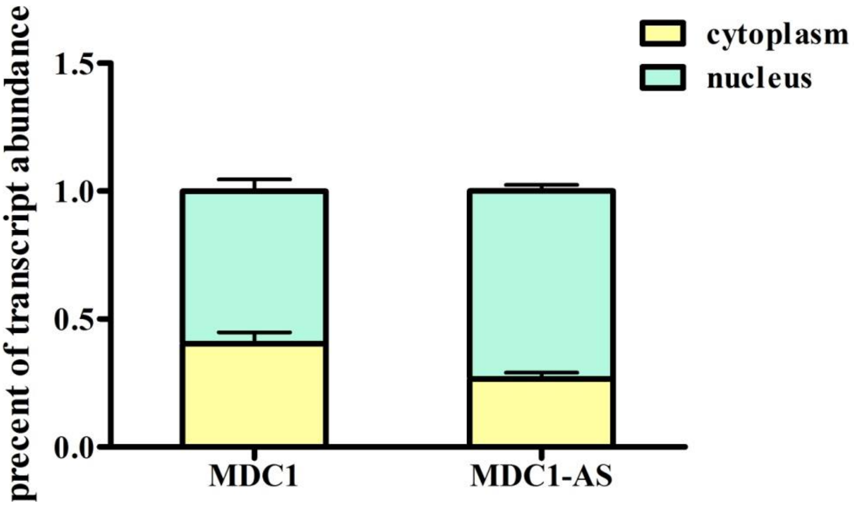

Supplementary Figure 2: Subcellular location analysis showed that both *MDC1-AS* and *MDC1* were exist primarily in the nuclear fraction.

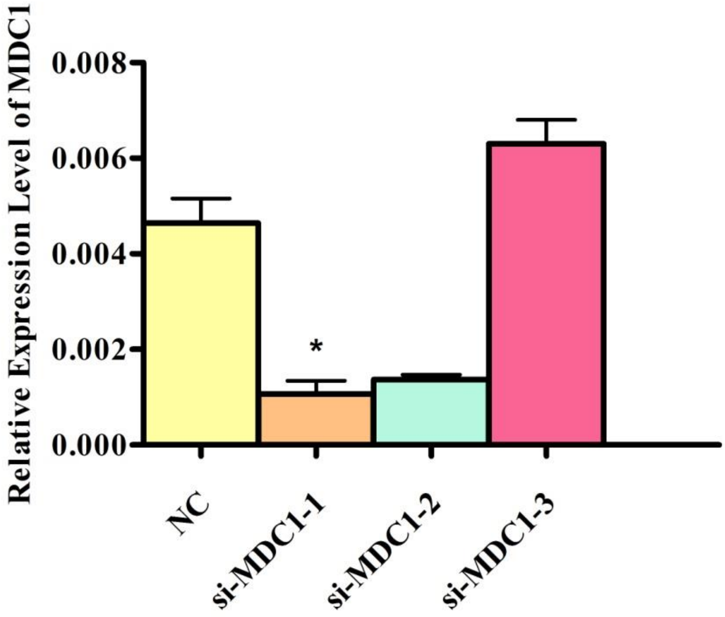

Supplementary Figure 3: Interfering efficiency of three si-MDC1 on knockdown of coding gene MDC1. Among them, si-MDC1-1 has the highest inhibition ratio.
